# Multi-layer molecular analysis reveals distinctive metabolomic and transcriptomic profiles of different sweet corn varieties

**DOI:** 10.3389/fpls.2024.1453031

**Published:** 2024-08-19

**Authors:** Kun Li, Jigang Zeng, Nan Zhang, Yongtao Yu, Wenguang Zhu, Gaoke Li, Jianguang Hu

**Affiliations:** ^1^ Guangdong Key Laboratory of Crop Genetic Improvement, Crop Research Institute, Guangdong Academy of Agricultural Sciences, Guangzhou, China; ^2^ College of Plant Science and Technology, Huazhong Agricultural University, Wuhan, Hubei, China

**Keywords:** sweet corn, sugar, transcriptome, metabolome, eGWAS

## Abstract

In plants, sugar metabolism involves a complex interplay of genetic, molecular and environmental factors. To better understand the molecular mechanisms underlying these processes, we utilized a multi-layered approach that integrated transcriptomic and metabolomic datasets generated from multiple different varieties of sweet corn. Through this analysis, we found 2533 genes that were differentially expressed in the immature kernel tissues of sweet corn, including genes involved in transcriptional regulation, sugar metabolism, primary metabolism, and other processes associated with adaptability of sweet corn. We also detected 31 differential metabolites among the three types of sweet corn. Utilizing an integrated approach encompassing transcriptomics and eGWAS, we elucidated the transcriptional regulatory patterns governing these differential metabolites. Specifically, we delved into the transcriptional modulation of malate- and ubiquitin-associated genes across a range of sweet corn varieties, shedding new light on the molecular mechanisms underlying their regulation. This study provides a framework for future research aimed at improving the current understanding of sugar metabolism and regulatory gene networks in sweet corn, which could ultimately lead to the development of novel strategies for crop improvement.

## 1 Introduction

Sweet corn (*Zea mays* L.) varieties contain mutations in genes associated with endosperm starch synthesis pathways, resulting in an abnormally high sugar accumulation (Tracy et al., 2019). The high sugar content of sweet corn has led to its popularity as a consumer crop (Revilla et al., 2021). Today the most common mutants used in commercial sweet corn varieties are the recessive gene *shrunken2* (*sh2*), followed by the combination of *sugary1* (*su1*) and *sugary1 enhancer1* (*su1-se1*) (Nelson and Pan, 1995; Zhang et al., 2019). S*h2*-based sweet corn varieties are known as super sweet corn and accumulate approximately six times more reducing sugars and sucrose in immature kernels than field corn (Chhabra et al., 2019). Sweet corn varieties based on the recessive gene *su1* accumulate approximately three times more reducing sugars and sucrose in their kernels at the milky ripening stage but when combined with *se1*, the water-soluble polysaccharides (WSP) levels are nearly as high as those of *sh2* (Tracy et al., 2019). There are also clear genetic boundaries that distinguish the differences in sugar metabolism, specifically, significant variations in genomic architecture among different types of sweet corn (Hu et al., 2021b).

Starch biosynthesis in maize is carried out through a series of enzyme reactions, with both protein metabolism and hormone regulation playing critical roles (Revilla et al., 2021). Sucrose is the main product derived from photosynthates that is utilized for long-distance transport from leaf tissue to developing kernels (Schleucher et al., 1998). In the kernel tissue, two enzymatic pathways are known to catalyze hydrolysis of sucrose (Kang et al., 2009). Cell wall invertase 2 (CWI2) breaks down sucrose into glucose and fructose, while sucrose synthase (SUS) converts sucrose to fructose and UDP-glucose, which are then used to synthesize cell wall polysaccharides with uridine diphosphate (UDP) (Winter and Huber, 2000; Koch, 2004). The different flavors and textures of sweet corn varieties may therefore arise from differences in these various sugar metabolism and transport pathways.

The low genetic diversity within each starch mutant has significantly hampered modern sweet corn breeding efforts (Revilla et al., 2021). Improving the diversity of different sweet corn varieties could therefore enable breeding efforts aimed at improving their taste and nutritional value (Mehta et al., 2020; Prasanna et al., 2020). For example, traditional super sweet corn kernels are deficient in some essential amino acids such as lysine, which could be attenuated by bringing more favorable alleles from field corn through marker assisted selection (MAS) (Mehta et al., 2020). A more comprehensive assessment of genetic diversity among sweet corn varieties could dramatically accelerate this process (Hu et al., 2021a; Xiao et al., 2022a).

Comprehensive analyses of multi-layer molecular data have been used to elucidate molecular mechanisms of complex traits. In recent years, applying integrated approaches using multi-omics data, such as metabolomics and transcriptomics, to various grass plants has proven to be an excellent method for analyzing sugar metabolic pathways and identifying regulatory genes (Tohge et al., 2005). For example, high temperatures have been shown to affect the metabolism and accumulation of sugars and starches in rice seeds, which may be related to changes in the expression of genes associated with sucrose, starch, and respiratory chains (Yamakawa and Hakata, 2010). A recent comparative analysis of metabolites related to appearance and taste in various tomato varieties has led to the identification of specific loci or genes that govern the reduction of antinutritional compounds (Zhu et al., 2018). Omics data has also recently been successfully applied to sweet corn research. For example, sweet corn seed quality, folates, vitamin E and carotenoid accumulation in the sweet corn kernels have all been studied using these techniques (Xiang et al., 2019; Xiao et al., 2022a, 2022b). However, there are few reports on the comparison of different sweet corn types at the transcriptomic and metabolomic levels, but this approach has the potential to provide novel avenues to explore for sweet corn improvement.

In this study, we assessed the transcriptomes and metabolomes of immature kernel tissues (including ordinary sweet corn, super sweet corn and enhanced sweet corn) at 15 and 20 days after pollination (DAP). We then utilized this data to explore changes in gene expression and metabolite profiles that were associated with disruption of starch synthesis. We also used transcriptome data obtained from a sweet corn population to conduct expression GWAS (eGWAS) of key genes related to malic acid and ubiquitin. By conducting a comparative analysis of multi-omics data, we obtained a more complete picture of the differences among sweet corn varieties. This understanding is crucial for the advancement of breeding practices in sweet corn.

## 2 Materials and methods

### 2.1 Sweet corn materials

We obtained 24 different corn varieties, including 8 ordinary sweet corn (*su1*), 8 enhanced sweet corn (*su1-se1*) and 8 super sweet corn (*sh2-R*) (Xiao et al., 2022a). The sweet corn material was planted in the experimental field of Guangdong Academy of Agricultural Sciences (113°22'E, 23°9'N), according to randomized block designs. All sweet corn experimental materials were self-pollinated, and immature kernel tissues were sampled at 15 and 20 days after pollination for transcriptome sequencing and metabolome identification, respectively. Transcriptome sequencing was performed on the immature kernels at 15 days after pollination (DAP), while metabolome identification was carried out on the kernels at 20 DAP. After sampling, sweet corn kernel tissues were frozen in liquid nitrogen immediately, then transferred to a -80°C freezer for storage.

### 2.2 Transcriptome data collection and expression assessment

Sweet corn transcriptome data of 24 samples generated in previous studies were downloaded from the China National GeneBank Database (CNGB, db https://db.cngb.org/, CNP0003294; Xiao et al., 2022a; **Supplementary Table S1**). RNA sequencing data of knockout lines of gene *sh2*, *su1* and wild types have been deposited into CNGB with the following accession number of CNP0003291. All transcriptome data were sequenced by BGI using PE150 mode. The transcriptomes of sweet corn were sequenced using the Illumina HiSeq TM 2500. All raw sequencing data were evaluated for expression abundance using the same analytical process. First, trimmomatic (v0.39) was used to filter the raw transcriptome data, including removing reads containing adaptors, low-quality reads, and reads with a proportion of N greater than 10% (Bolger et al., 2014). All clean reads were then mapped to the B73 reference genome (AGP v4) using HISAT2 (Version v2.2.1) (Pertea et al., 2016). After mapping, SAMtools (Version v1.17) was used to convert and compress the intermediate data (Li et al., 2009). Finally, the gene expression values were quantified by the FeatureCounts tool in Subread (Version 2.0.3) (Liao et al., 2013, 2014).

### 2.3 Differentially expressed gene identification

Differentially expressed genes (DEGs) were calculated with DESeq2 (Version 1.36.0) performed in the R (Version 4.3.0) environment (Love et al., 2014). The raw expression count of each gene with expression values greater than 10 were used as inputs to DESeq2. The q-value of each gene was calculated from corrected p-values with the Benjamini-Hochberg multiple hypothesis test correction model. The threshold for differentially expressed genes was q-value<0.05 and fold change greater than 2.

### 2.4 Metabolite extraction and GC-MS based metabolomic analysis

Metabolomic data from 24 sweet corn lines were obtained from our previously study (Xiao et al., 2022a). For this experiment, immature sweet corn kernels (20 DAP) were harvested with two biological replicates. Samples were then ground with a mill (MM400; Retsch) in a pre-cooled environment with liquid nitrogen. Fifty milligrams of sample powder was then utilized for metabolite extraction, according to methods described in previous studies (Salem et al., 2016; Wang et al., 2019). After suspension, 200 μL of the lower phase were transferred into a 1.5 mL microcentrifuge tube, then centrifuged at 14000 rpm for 10 min at 4°C. The samples were dried with a SpeedVac concentrator at 25°C. Prior to GC-MS (7890A-5975C, Agilent, USA), the samples were derivatized with N-methyl-N-(trimethylsilyl) trifluoroacetamide, as described previously (Yan et al., 2018). One μL of each sample was injected into the GC-MS instrument with a DB-35MS UI (30 m × 0.25 mm, 0.25 µm) capillary column in split mode at 270°C. The flow of helium carrier gas was set to 1 mL/min and the mass range of samples analyzed was from m/z 85 to 700. Agilent MassHunter Qualitative Analysis software version B06.00 and B.07.01 were jointly used for data analysis. NIST library and an in-house database constructed with authentic standards were utilized together for metabolite identification.

### 2.5 Functional enrichment analysis and visualization

Gene Ontology analysis (GO, https://www.geneontology.org/) and Kyoto Encyclopedia of Genes and Genomes (KEGG, https://www.genome.jp/kegg/) pathway enrichment analysis were conducted using the package clusterProfiler (v3.8.1) (Yu et al., 2012) and visualized via the package ggplot2 (v3.0.0) in the R environment. The significantly enriched GO terms and KEGG categories were selected with Benjamini–Hochberg adjusted P-values (FDR) <0.05.

### 2.6 Starch content determination

Starch can form a complex with iodine and exhibit a distinctive color. Amylopectin forms a brown-red complex with iodine, while amylose forms a dark blue complex with iodine. The absorption values of amylose and amylopectin at wavelengths λ1 and λ2 were determined by dual-wavelength spectrophotometry (Li et al., 2016; Han et al., 2019). The starch content of sweet corn kernels was calculated according to the linear relationship between ΔA (ΔA=Aλ1-Aλ2) and starch concentration. Each sample was ground, and 100 mg was added to 1 mL of absolute ethanol and 9 mL of 1 mol/L NaOH. The sample was then dissolved in a boiling water bath for 10 min. The sample extract was degreased with petroleum ether two to three times. Then, 3 mL of the degreased sample was measured to determine the absorbance and calculate the corresponding starch content via the standard linear equation.

### 2.7 Weighted gene correlation network analysis

A weighted gene correlation network analysis (WGCNA) was conducted based on the 2533 differentially expressed genes in the sweet corn kernel tissues (Langfelder and Horvath, 2008). The network was generated based on Spearman correlations among genes and clustered into modules with a dynamic tree cut model with a deep split level of 3 and a height of 0.25. Finally, the eigenvector and eigengene of each module was calculated as the representative value of the module. The correlations within modules and between modules and phenotypes were then utilized for downstream analyses. To validate the stability and reliability of the network, we calculated the Zsummary, a module size-dependent robustness parameter that considers both module density preservation and connectivity preservation (Langfelder et al., 2011). This was done with 20 times random resampling of the RNA-seq datasets in each genotype. Modules with a Zsummary greater than 10 implied strong module preservation, indicating a high degree of confidence in the data. Modules with a Zsummary greater than 2 were considered stable and reliably associated with metabolites.

### 2.8 Expression genome-wide association study

Gene expression genome-wide association was conducted according to our previous reports (Xiao et al., 2022a). The association analysis was conducted using Tassel (version 3.0) with a mixed linear model (MLM), which controls for cofactors of population structure (Q) and kinship matrix (K). The threshold of association analysis was determined with GEC (Genetic Type I error calculator; (Li et al., 2012). Specifically, we used the formula P-value = 1/n, where n represents the effective number of independent SNPs. In our study, 9,858,639 SNPs were used for the evaluation of the effective number, resulting in a calculation of 1,970,123 as the effective number of independent SNPs. Consequently, the significance threshold for the sweet corn population was set at approximately P=5.08×10^-7^. Candidate genes were selected based on the position of the lead SNP, which was located within each significant QTL. Genes within a 500 kb region upstream or downstream of this position were considered candidate causal genes.

## 3 Results

### 3.1 Sweet corn kernel appearances and sugar contents

**Figure 1:**
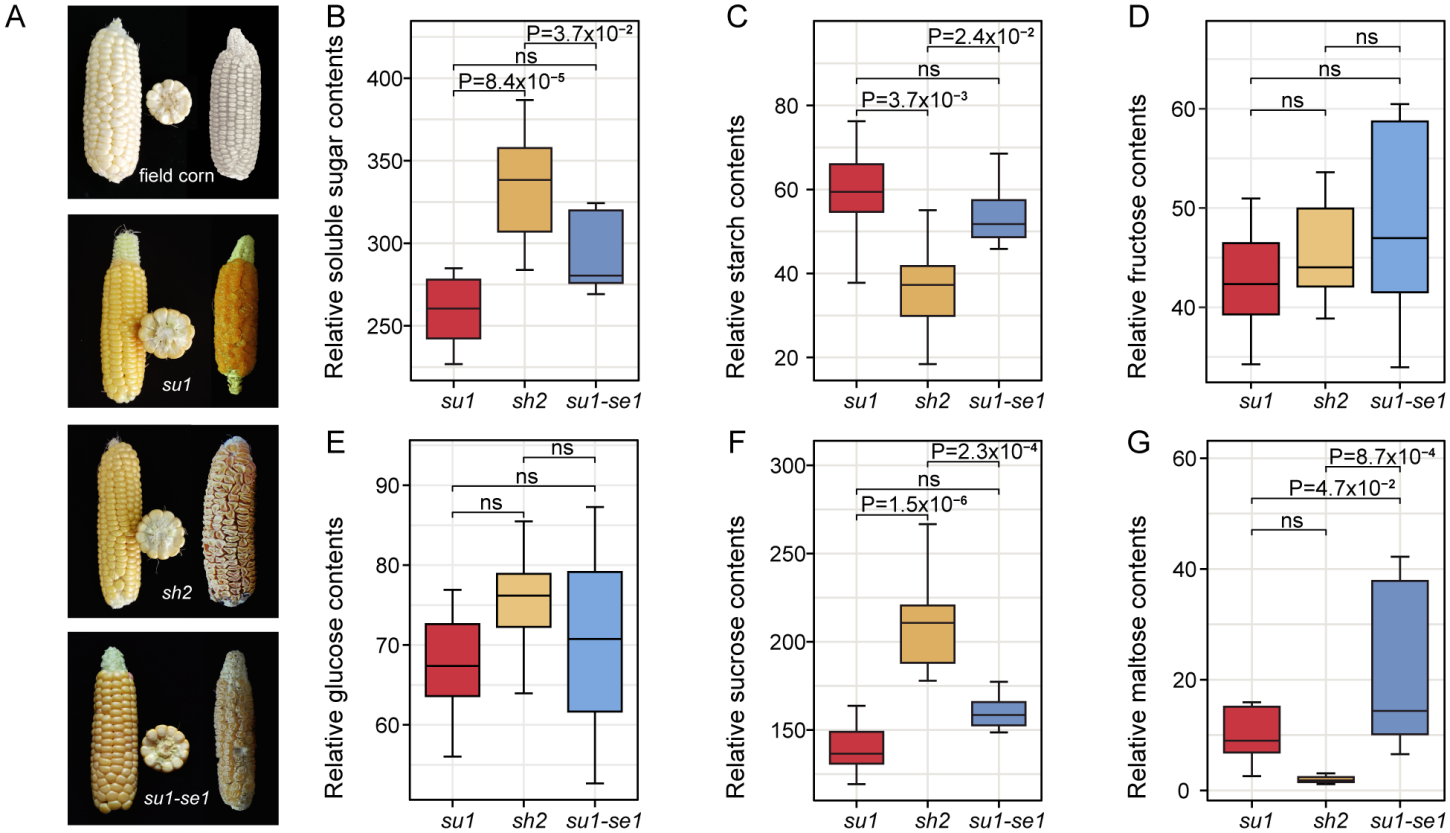
Appearance and sugar content of sweet corn kernels. **(A)** Appearance of fresh and dried ears of four types of corn. **(B–G)** Box plots of soluble sugar, starch, fructose, glucose, sucrose, and maltose contents of 24 sweet corn individuals, respectively. ns, not significant.

Different types of sweet corn exhibit significant differences in kernel appearance, likely due to variations at the transcriptomic and metabolomic levels (**Figure 1A**). The dry kernels of *sh2* sweet corn display shriveled and sunken features with reduced luster compared to field corn kernels (**Figure 1A**). We used R software for variance analysis and TukeyHSD multiple tests to compare the differences in soluble sugar (including fructose, glucose, sucrose, and maltose) and starch content in the fresh kernel tissue of sweet corn varieties (**Figures 1B–G**). Although fructose and glucose exhibited a high degree of phenotypic variation, there were no significant differences observed between different sweet corn varieties, possibly due to the minimal impact of genetic background on these two monosaccharides (Harakotr et al., 2022). Furthermore, there were notable disparities in sucrose, maltose, and starch content, with the most pronounced differences observed in super sweet corn (*sh2*) kernels (**Figures 1C, F, G**). The variations between ordinary sweet corn (*su1*) and enhanced sweet corn (*su1-se1*) kernels were primarily attributed to differences in maltose content (**Figure 1G**). The super sweet corn kernels had 1.29 and 1.20 times the level of soluble sugar found in ordinary sweet corn and enhanced sweet corn kernels, respectively (**Figure 1B**). Notably, super sweet corn kernels had the highest sucrose content, which was 1.54 and 1.33 times that of ordinary sweet corn and enhanced sweet corn kernels, respectively (P=2.23×10^-4^). There were also significant disparities in maltose content among the three sweet corn types, with enhanced sweet corn kernels containing 7.89 and 1.60-fold more maltose than super sweet corn and ordinary sweet corn kernels, respectively (**Figure 1G**). This indicates that the enhanced activity of starch-degrading enzymes in enhanced sweet corn result in the breakdown of starch into maltose (Zhang et al., 2019). Ordinary sweet corn kernels had the highest starch levels, which were 1.59 and 1.14 times those of super sweet corn and enhanced sweet corn kernels, respectively (**Figure 1C**). The super sweet corn kernels had the highest average amounts of soluble sugars, glucose, and sucrose, while enhanced sweet corn kernels had the highest average quantities for fructose and maltose.

### 3.2 Differentially expressed genes in sweet corn kernels

**Figure 2:**
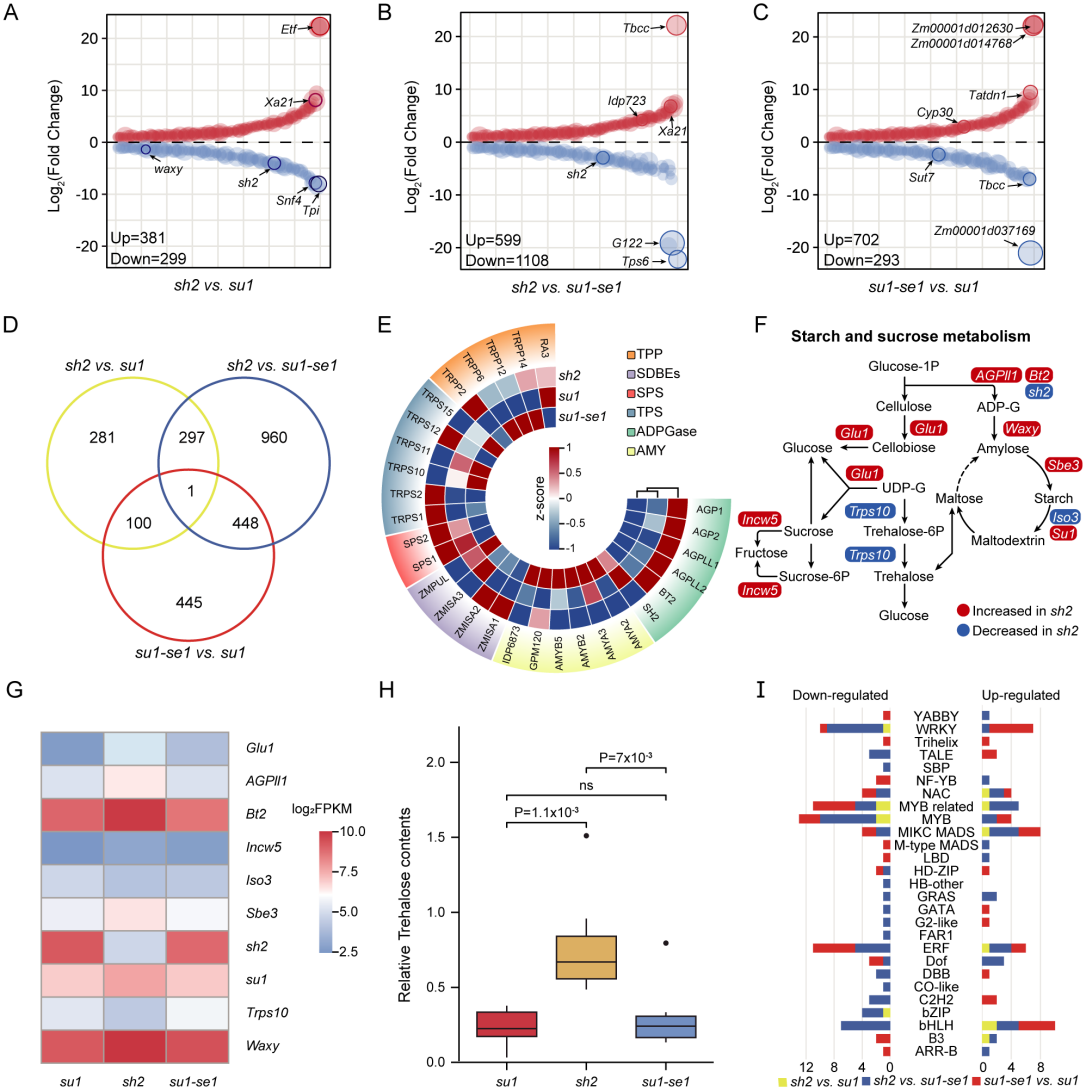
DEGs obtained from comparisons of the three types of sweet corn kernels. **(A–C)** DEGs obtained from the comparison of *sh2* versus *su1, sh2* versus *su1-se1* and *su1-se1* versus *su1*. **(D)** Venn diagram of DEGs identified from the three types of sweet corn kernels. **(E)** Expression levels of genes encoding key enzymes in the starch synthesis pathway. **(F)** Pathways related to starch and sucrose synthesis and metabolism. **(G)** Expression levels of genes related to starch synthesis and metabolism. **(H)** Trehalose contents of the three types of sweet corn. **(I)** Statistics of differentially expressed genes of 27 transcription factor families. ns, not significant.

We performed transcriptome sequencing on 24 sweet corn kernel samples, resulting in a total of 289.2 million reads (**Supplementary Table S1**). After alignment to the B73 reference genome (v4.0), 28,698 genes were found to have detectible expression. Differential expression analysis was carried out using the DESeq2 package, with a threshold of |log_2_foldchange| > 1 and q-value < 0.05. Finally, a total of 2533 differentially expressed genes (DEGs) were identified (**Figures 2A–C**; **Supplementary Table S2**). There were 680, 1707, and 995 DEGs identified in the comparisons of *sh2* versus *su1*, *sh2* versus *su1-se1*, and *su1-se1* versus *su1*, respectively. Of them, a total of 285 DEGs were identified across the comparisons between the knockout lines of *sh2* and *su1* and their respective wild types (**Supplementary Table S2**). Among them, the number of down-regulated DEGs in the comparison of *sh2* versus *su1-se1* accounted for 43.74% of the total DEGs, while the number of up-regulated DEGs in the comparison of *su1-se1* versus *su1* was the lowest, accounting for 11.57% of the total DEGs (**Figure 2D**).

Examination of 29 genes known to be in sucrose and starch synthesis pathways revealed the highest level of differential expression in comparisons of super sweet corn to ordinary sweet corn or enhanced sweet corn (**Figure 2E**). In super sweet corn, most DEGs in the starch and sucrose metabolic pathways were elevated compared to other varieties, including granule-bound starch synthase1 (*Waxy1*, Zm00001d033937), AGPase small subunit glucose-1-phosphate adenylyltransferase small subunit2 (*Bt2*, Zm00001d032385), starch debranching enzyme1 (*Su1*, Zm00001d049753), starch branching enzyme3 (*Sbe3*, Zm00001d011301), two glycoside hydrolases glucosidase1 (*Glu1*, Zm00001d023994) and invertase cell wall5 (*Incw5*, Zm00001d025354), and one glycosyltransferase ADP glucose pyrophosphorylase large subunit leaf1AGPL4 (*Agpii1*, Zm00001d033910). Down-regulated genes included starch debranching enzyme isoamylase-type starch debranching enzyme3 (*Iso3*, Zm00001d020799), which is involved in starch degradation, and glycosyltransferase trehalose-6-phosphate synthase10 (*Trps10*, Zm00001d052060), which is involved in trehalose synthesis (**Figures 2F, G**). Additionally, *Trps10*, which is known to affect starch content and grain weight, was found as a putative causal gene located in a QTL for total starch content (Hu et al., 2021b). This gene also exerts a significant effect on total trehalose content (**Figure 2H**).

In addition to structural genes involved in starch and sugar metabolic pathways, we also identified a significant number of regulatory genes that were differentially expressed in sweet corn, including several transcription factors. The transcription factors identified play a diverse array of roles, including not only regulation of sugar metabolism but also primary metabolism and stress signaling (**Figure 2I**; **Supplementary Table S2**). For example, in the comparison of *sh2* versus *su1*, the MYB-related transcription factor family had the most differential expression, with down-regulated transcription factor MYBR13 (Zm00001d038288) being a direct downstream gene of opaque endosperm2 (*O2*, Zm00001d018971) (Li et al., 2015), which is known to have a synergistic effect with NAC transcription factors 130 (*ZmNAC130*, Zm00001d008403) and *ZmNAC128* (Zm00001d040189) in promoting maize endosperm filling (Chen et al., 2023). In the comparison of *sh2* versus *su1-se1*, the bHLH family was the most up-regulated transcription factor family, which is known to participate in various physiological processes that impact plant growth and development (Zhang et al., 2018). The WRKY family was the most downregulated transcription factor family and is known to play a role in numerous biological processes and stress responses in plants (Zhang et al., 2017). In the comparison of *su1-se1* versus *su1*, the *MIKC MADS* family had the most up-regulated genes, and this gene family plays an important role in flower meristem formation and flower organ development (Gramzow and Theißen, 2013). The ERF family, which controls several stress responses and plays an important role in integrating sugar, ABA and ethylene signaling, contained five downregulated genes (Zhou et al., 2012; Finegan et al., 2022).

### 3.3 Functional enrichment analysis of differentially expressed genes

**Figure 3:**
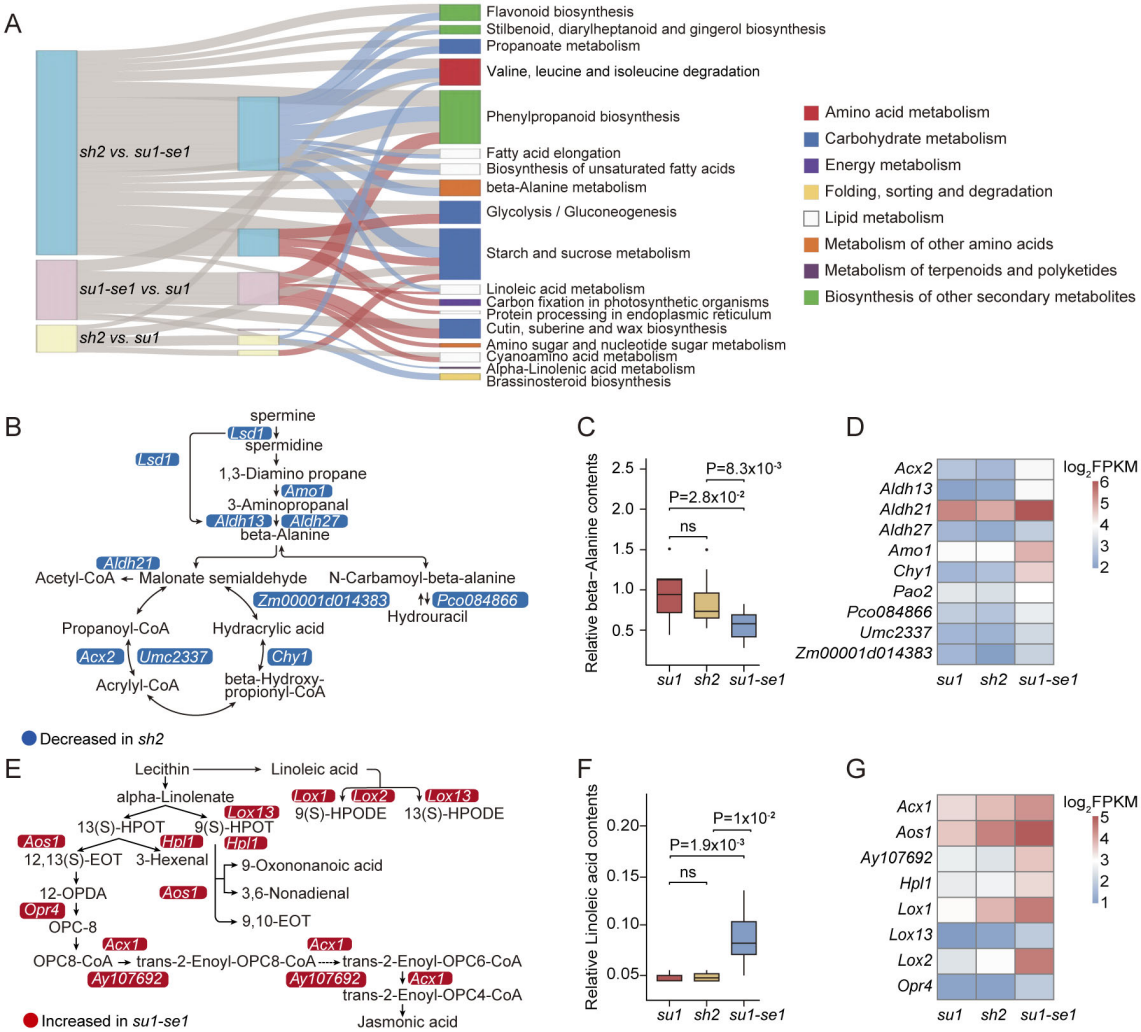
Functional analysis of DEGs. **(A)** Sankey diagram showing KEGG enrichment of DEGs. **(B)** Schematic diagram of beta-alanine related pathways. **(C)** Gene expression levels of key genes related to beta-alanine pathways. **(D)** Boxplot of beta-alanine contents of the three types of sweet corn. **(E)** Schematic diagram of linoleic acid-related pathways. **(F)** Gene expression levels of key genes related to linoleic acid pathways. **(G)** Boxplot of linoleic acid contents of the three types of sweet corn. ns, not significant.

To investigate the biological functions of differentially expressed genes (DEGs), we conducted enrichment analysis of DEGs, including Kyoto Encyclopedia of Genes and Genomes (KEGG) and Gene Ontology (GO) enrichment analysis. In the comparison of *sh2* and *su1*, DEGs were enriched in 2 KEGG pathways, including starch and sucrose metabolism, as well as degradation pathways of valine, leucine, and isoleucine (q-value <0.05; **Figure 3A**; **Supplementary Table S3**). In the comparison between *sh2* and *su1-se1*, DEGs were enriched in 13 KEGG pathways, with a significant enrichment in the β-alanine metabolism pathway (q-value <0.05; **Figure 3A**; **Supplementary Table S3**). In the comparison between *su1-se1* and *su1*, DEGs were enriched in 3 KEGG pathways, including α-linolenic acid metabolism and the linoleic acid pathway (q-value <0.05; **Figure 3A**; **Supplementary Table S3**). These results indicate significant differences in gene transcription regulation between different types of sweet corn, not only in sugar metabolism-related pathways, but also in amino acid and ester metabolism. GO analysis showed that differentially expressed genes were significantly enriched in metabolic pathways related to sugar metabolism, as well as other metabolic pathways, such as photosynthesis, protein synthesis, citric acid metabolism, cellular lipid metabolism, and organic acid biosynthesis (q-value <0.05; **Supplementary Table S4**). The analysis of these regulatory patterns of metabolic pathways provides a deeper and more comprehensive understanding of the gene regulatory network in sweet corn.

In the *sh2* versus *su1-se1* comparison, DEGs involved in β-alanine metabolism were generally down-regulated, including a dihydropyrimidine dehydrogenase (*Pco084866*, Zm00001d043152), a hydrolyase 3-hydroxyisobutyryl-CoA hydrolase1 (*Chy1*, Zm00001d023779), three annotated aldehyde dehydrogenases (*Aldh13*, Zm00001d004731; *Aldh21*, Zm00001d019054; *Aldh27*, Zm00001d025958), and four oxidoreductases (Zm00001d014383; *Umc2337*, Zm00001d045606; *Lsd1*, Zm00001d035195; *Amo1*, Zm00001d025103; *Acx2*, Zm00001d042884) (**Figure 3B**). There is evidence that β-alanine is involved in lignin biosynthesis and ethylene production in some species (Parthasarathy et al., 2019), and there was a significant negative correlation between alanine content and the expression of genes in its metabolic pathway in sweet corn kernels. The alanine content in enhanced sweet corn was significantly lower than that in the other two types, while there was no significant difference between ordinary sweet corn and super sweet corn (**Figures 3C, D**; **Supplementary Table S5**). In the *su1-se1* versus *su1* comparison, all DEGs involved in α-linolenic acid and linoleic acid metabolism were up-regulated, including four lipoxygenases (*Lox1*, Zm00001d042541; *Lox2*, Zm00001d042540; *Lox8*, Zm00001d003533; *Lox13*, Zm00001d031449), one prostaglandin synthase alternative oxidase1 (*Aox1*, Zm00001d017727), one hydroperoxide lyase (*Hpl1*, Zm00001d054067), one 12-oxophytodienoic acid reductase4 (*Opr4*, Zm00001d011097), one acyl-CoA oxidase (*Acx*, Zm00001d045251), and one peroxidase (*Ay107692*, Zm00001d048890) (**Figure 3E**). The average expression levels of genes in the α-linolenic acid and linoleic acid metabolic pathways of sweet corn grains of *su1-se1* were higher than other sweet corn varieties, with a resultant increase in linoleic acid content (**Figures 3F, G**). Since α-linolenic acid and linoleic acid are polyunsaturated fatty acids, their metabolism in plants is closely related to plant growth, development and stress response (Grechkin, 1998; Baldwin et al., 2006; Matsui, 2006; Truman et al., 2007; Borrego and Kolomiets, 2016). This result indicates that *su1-se1* sweet corn kernels may have more active metabolic activities in these two fatty acid synthesis pathways, leading to an increase in fatty acid content. This finding has important implications for the regulatory mechanisms underlying *su1-se1* sweet corn phenotypes and provides new avenues of research into other key metabolic pathways.

### 3.4 Metabolic profile analysis of sweet corn kernels

We utilized chromatography-mass spectrometer (GC-MS) to identify and quantify 223 metabolites in the kernel tissue of 24 sweet corn individuals, including sugars, amino acids, lipids, flavonoids, and other small molecules (**Supplementary Table S5**). Lipid molecules mainly included isoprenoid lipids and fatty acids. Organic acids were mainly comprised of carboxylic acids and their derivatives, while phenylpropanoids and polyketides mainly included flavonoids, isoflavonoids, coumarins, cinnamic acid and their derivatives. The organic oxygen compounds identified were primarily indoles and their derivatives.

**Figure 4:**
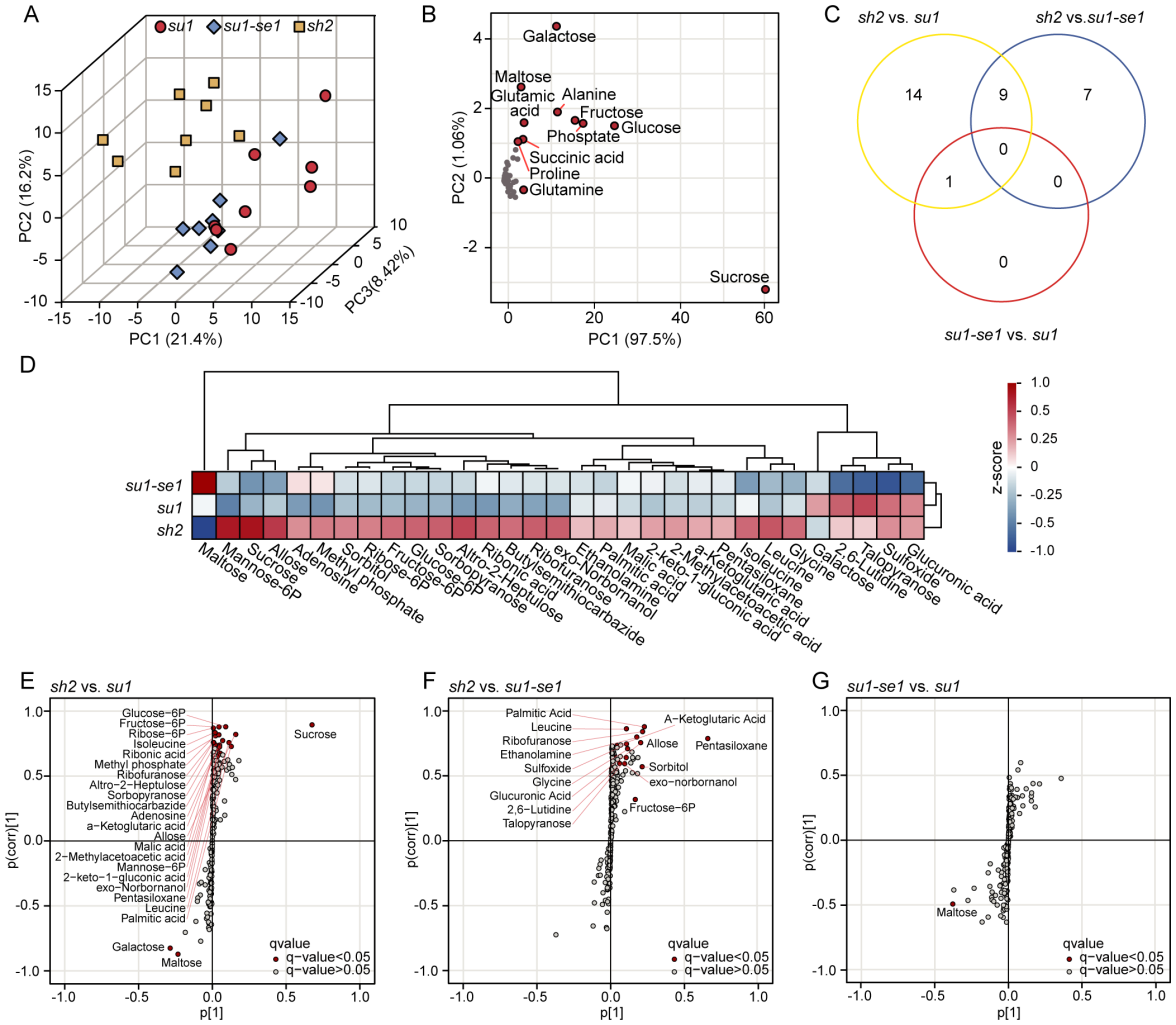
Identification of differentially expressed metabolites in the three types of sweet corn. **(A)** Principal component analysis of sweet corn individuals based on metabolome data. **(B)** Principal component analysis of metabolome data. **(C)** Venn diagrams of differentially expressed metabolites identified from three comparisons. **(D)** Cluster analysis of differentially expressed metabolites. E-G. S-plot from OPLS-DA analysis showing the distribution of metabolites based on their covariance (x-axis) and correlation (y-axis) with the predictive component. The plot highlights metabolites that are significantly different between the experimental groups, with red points indicating metabolites having VIP (Variable Importance in Projection) scores greater than 1 and q-value<0.05. This analysis compared *sh2* with *su1*, *sh2* with *su1-se1*, and *su1-se1* with *su1*.

A PCA analysis revealed that different types of sweet corn had different metabolic profiles (**Figure 4A**). The metabolic profiles of super sweet corn, ordinary sweet corn, and enhanced sweet corn differed significantly, while the difference between the metabolic composition of *su1* and *su1-se1* kernel tissues was relatively small (**Figure 4A**). Replicate samples had highly similar metabolic profiles and clustered together. PC1, PC2, and PC3 jointly accounted for 46.02% of the variance in metabolic content (**Figure 4A**). Further analysis of the PCA indicated that most metabolites had a similar distribution pattern, with significant clustering (**Figure 4B**). PC1 and PC2 explained 98.56% of the variance, while a few substances, such as sucrose, glucose and fructose alanine had distinct profiles in sweet corn, resulting in a clear separation within the metabolic grouping data (**Figure 4B**). Among them, sucrose had the greatest difference in three types of sweet corn (**Figure 4B**).

Difference orthogonal partial least squares discriminant analysis (OPLS-DA) has been demonstrated to be useful for the identification of differential metabolites between different types of sweet corn (Bylesjö et al., 2006). An analysis of differential metabolites was carried out for three comparisons: *su1-se1* versus *su1*, *sh2* versus *su1-se1*, and *sh2* versus *su1*, resulting in the identification of 31 differential metabolites (VIP>1, q-value<0.05). Twenty-four metabolites were significantly changed in *sh2* versus *su1*, 16 in *sh2* versus *su1-se1*, and 14 in *su1-se1* versus *su1* (**Figure 4C**; **Supplementary Table S6**). To further assess the distribution of metabolites in the kernel tissues of different sweet corn varieties, hierarchical clustering was carried out on the 31 differential metabolites. Super sweet corn had higher levels of most of the differential metabolites, including lysine and other substances. However, maltose content was lowest in super sweet corn and highest in enhanced sweet corn. Talopyranose and galactose content was highest in ordinary sweet corn (**Figure 4D**).

S-plot analysis can be used to reveal the covariance between metabolites and the predictive component as well as correlation coefficient (Wiklund et al., 2008; Pan et al., 2021). In the comparison of *sh2* versus *su1*, the differential metabolites maltose and isomaltose were positively correlated, while sucrose was negatively correlated with these metabolites. In the comparison of the *sh2* versus *su1-se1*, the differential metabolites leucine and glutamine were positively correlated, while maltose was negatively correlated with these metabolites. In the comparison of the *su1-se1* versus *su1*, the differential metabolites alanine and glucose-6-phosphate were negatively correlated (**Figures 4E–G**). These results suggest that different sweet corn varieties have different metabolite accumulation and conversion patterns in their kernel tissues during the milk stage (Ferguson et al., 1979; Venkatesh et al., 2016), which may be related to differences at the transcriptional level that affect sugar accumulation and conversion (Zhang et al., 2019; Finegan et al., 2022; Harakotr et al., 2022).

### 3.5 Weighted co-expression network analysis

**Figure 5:**
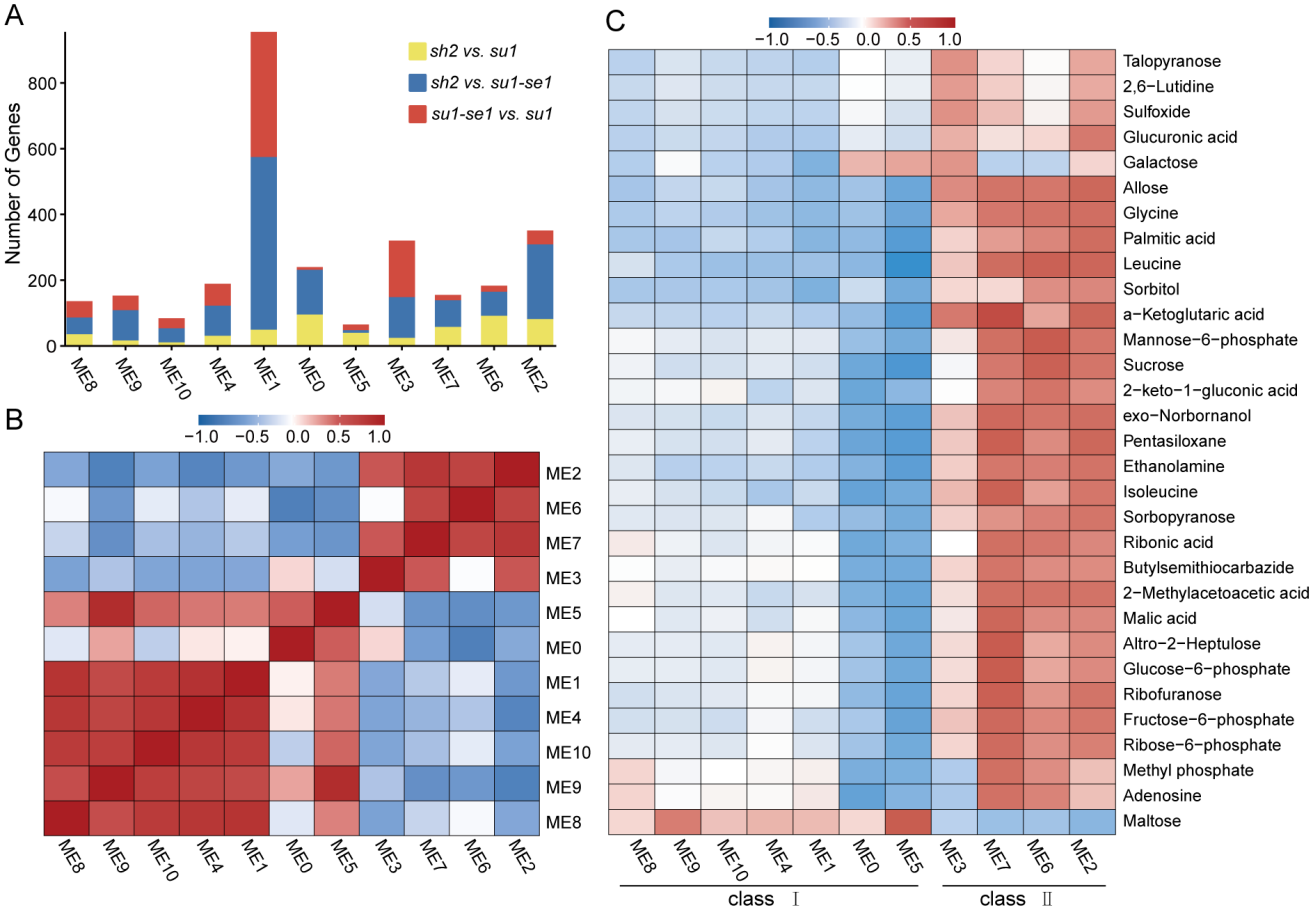
Weighted correlation network analysis of DEMs and transcriptome data. **(A)** DEGs distribution in the modules identified by WGCNA. **(B)** Correlation analysis of modules in WGCNA. **(C)** Correlation analysis of DEMs and DEG modules.

We employed weighted gene co-expression network analysis to gain insights into the transcriptional patterns of genotypes and unravel the associations between distinct modular expression patterns and metabolite levels. Utilizing the WGCNA package in R software (Langfelder and Horvath, 2008), we grouped 2533 DEGs into 11 co-expression modules, with module sizes ranging from 89 to 796 genes and averaging at 241 genes per module (**Figure 5A**; **Supplementary Table S7**). The genes within each module displayed robust connectivity (Z-value>2) (Langfelder et al., 2011). Correlation analysis among modules revealed a rough segregation into two major classes, characterized by strong positive correlations within each class and negative correlations between classes (**Figure 5B**). Among the 31 differential metabolites analyzed, class 1 had a negative correlation with the majority of the other metabolites, while class 2 exhibited a positive correlation with most differential metabolites (**Figure 5C**). Interestingly, maltose content was positively correlated with class 1 but negatively correlated with class 2 (**Figure 5C**). In line with these findings, maltose content was found to be notably elevated in enhanced sweet corn compared to other sweet corn varieties, whereas most differential metabolites were most enriched in super sweet corn (**Figure 5C**).

**Figure 6:**
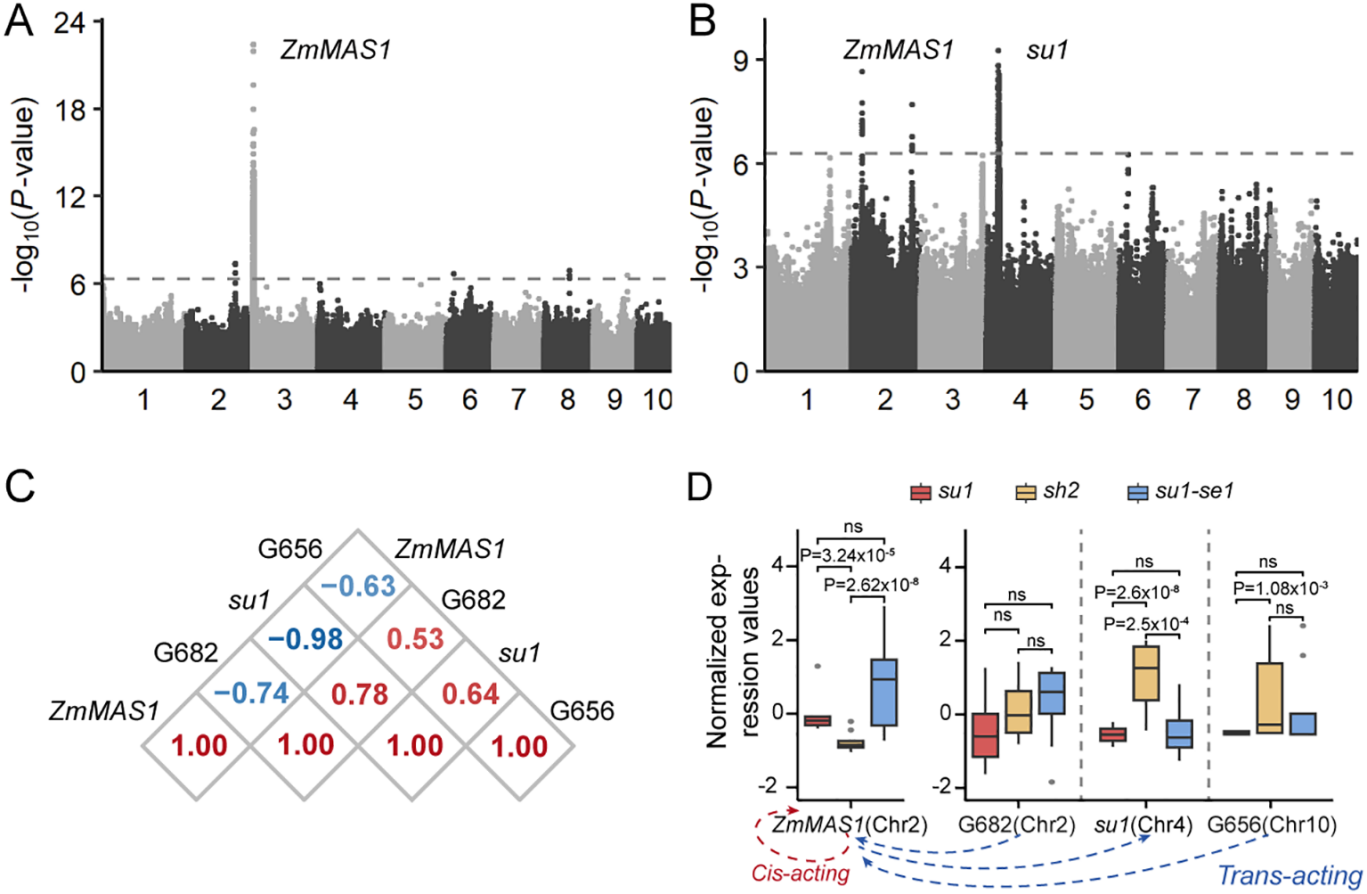
Transcriptional regulation of genes involved in malic acid pathways. **(A)** Expression GWAS of *ZmMAS1*. **(B)** Expression GWAS of *Zm00001d023656*. **(C)** Correlation analysis of expression values of genes in the regulatory network of *ZmMAS1*. **(D)** Expression values of genes related to the *ZmMAS1* regulatory network among three types of sweet corn (upper) and regulatory network related to *ZmMAS1*(lower). G656, Zm00001d023656; G682, Zm00001d006682. ns, not significant.

Genes contained within the same regulatory network frequently exhibited tightly correlated expression patterns, mirroring their interconnected biological functions. For instance, the gene ZmMAS1 (Zm00001d003247), located within module 5, encodes malate synthase and had a marked positive correlation in expression with the metabolite malic acid (**Figure 5C**). A comprehensive genome-wide association study of expression levels of ZmMAS1 revealed that this gene was not solely influenced by its own cis-eQTL but was also modulated by two distinct trans-eQTL loci encoding the genes *Su1* on chromosome 4 and Zm00001d023656 on chromosome 10 (**Figures 6A, B**). Furthermore, the *ZmMAS1* locus was found to regulate the expression of Zm00001d023656 via a trans-eQTL (**Figure 6C**). These four genes, three of which were found to be differentially expressed, are integral players in the metabolic regulation of sweet corn (**Figure 6D**).

**Figure 7:**
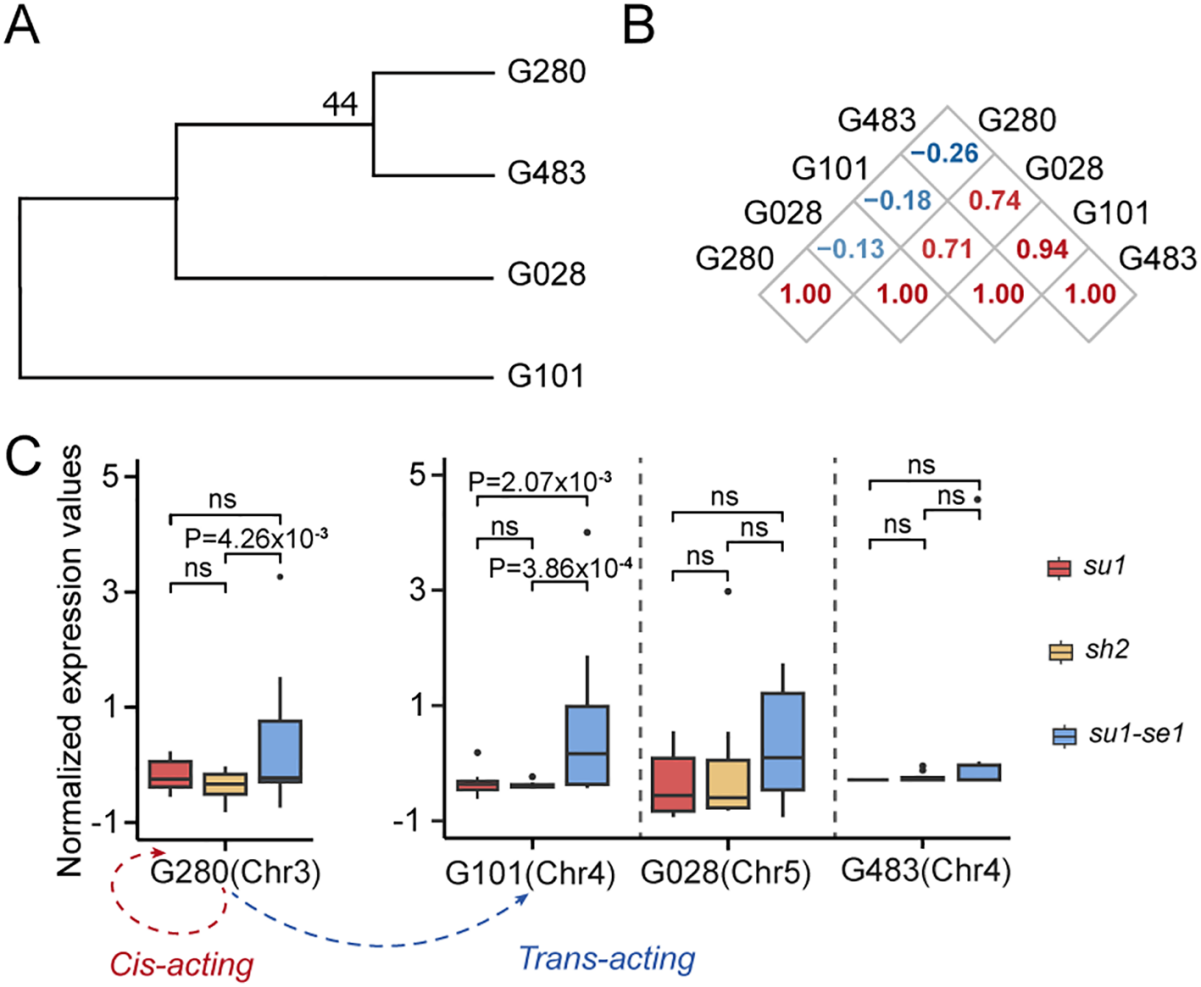
Transcriptional regulation of genes related to ubiquitin. **(A)** Phylogenetic tree of genes related to ubiquitin. **(B)** Correlation analysis of expression of genes that are homologous to Zm00001d049101. **(C)** Expression values of genes that are homologous to Zm00001d049101 among three types of sweet corn (upper) and regulation network related to Zm00001d049101 (lower). G483, Zm00001d051483; G101, Zm00001d049101; G028, Zm00001d015028; G280, Zm00001d039280. ns, not significant.

Protein ubiquitination is a ubiquitous post-translational modification that controls the turnover of a diverse array of proteins and serves as a pivotal regulatory mechanism for plant biological processes. Our analysis revealed a significant correlation in the expression levels of two homologous genes encoding ubiquitin enzymes, Zm00001d039280 and Zm00001d049101 (**Figures 7A, B**). An in-depth expression-level association study indicated that the gene Zm00001d039280 not only auto-regulates its own expression but also modulates the expression of Zm00001d049101 via a trans-eQTL (**Figure 7C**). This regulatory interplay results in differential expression patterns across various sweet corn varieties, ultimately influencing the development of distinct sweet corn types.

## 4 Discussion

### 4.1 Significant variations exist in the composition of sweet corn

Ordinary sweet corn, enhanced sweet corn and super sweet corn kernels have been found to be composed of approximately 24%, 14% and 7% starch, respectively (Szymanek et al., 2015). In addition to differing starch content, the three sweet corn varieties also show variable transcriptomes, metabolomes and stress responses (Silva et al., 2020; Stansluos et al., 2020). Genetic background, growth environment, and artificial selection all contribute to the different compositions of sweet corn kernels. Previous studies have shown that *sh2* is located upstream of the starch biosynthesis pathway, while *su1* works downstream of this pathway (Kramer et al., 2015). There are significant differences in the appearance of kernels among different types of sweet corn, with the kernels of super sweet corn exhibiting a shrunken appearance and decreased glossiness. These changes in appearance are likely related to the sugar accumulation and metabolism of sweet corn (**Figure 1**). There are also significant differences in the total soluble sugar, sucrose, and maltose content of different types of sweet corn, with *sh2* having especially high levels of soluble sugar and sucrose (**Figure 1**). The variance in sugar accumulation observed among distinct varieties of sweet corn can potentially be attributed to disparities in metabolome and transcriptional regulation.

There have been many reports on differences in sugar-related metabolites in different types of sweet corn, but metabolomics has rarely been employed in this area of research (Yang et al., 2020; Finegan et al., 2022; Rohilla and Singh, 2022). Significant differences in taste and texture exist among different sweet corn, which cannot be fully explained by variations in sugars alone (Tracy et al., 2019). In this study, metabolomic analysis revealed compositional differences among 223 metabolites in various types of sweet corn kernels. Principal component analysis (PCA) revealed distinct metabolite profiles among different types of sweet corn, with the most notable differences observed between super sweet corn and ordinary sweet corn (**Figure 4A**). Through DEMs analysis, we identified metabolites with significant differential accumulation across different sweet corn types, including glucose-6-phosphatase (G-6-P), gentiobiose, and β-alanine. The fluctuations in these metabolites are likely closely linked to the regulation of their gene expression levels, and result in differences in flavor profiles among sweet corn. Further, these differences impact a number of nutrients and metabolites, such as vitamin E and folate (Baseggio et al., 2019; Lv et al., 2022; Xiao et al., 2022a). These metabolites may also influence the nutritional quality of sweet corn, potentially affecting dietary intake of essential nutrients.

### 4.2 Transcriptional regulation plays a pivotal role in the development of sweet corn

Starch synthesis and metabolic pathways play major roles in plant development and any functional deficiency in these pathways can lead to significant alterations in overall transcriptional regulation (Finegan et al., 2022). Unsurprisingly, mutants deficient in these pathways exhibit significant differences in physiological responses and gene expression patterns (**Figure 2**). We employed transcriptome analysis to identify a large number of DEGs when comparing the kernels of different sweet corn types. The comparison of *sh2* versus *su1se1* revealed the most significant differences, indicating that there were complex regulatory changes occurring during the milk stage that impacted multiple aspects of sugar metabolism, growth, development and stress responses (**Figure 2B**).

Our analysis of DEGs uncovered several crucial transcription factor families that were differentially expressed in sweet corn, including MYB, bHLH, and WRKY, which play pivotal roles in plant growth, development, and metabolic regulation. Among these, genes associated with starch synthesis, such as alpha amylase3 (*Amya3*, Zm00001d005890) and pullulanase type starch debranching enzyme1 (*Pul*, Zm00001d004438), were significantly downregulated in super sweet corn kernels, potentially contributing to the lower starch content in sh2-type sweet corn. Furthermore, through GO and KEGG analyses we identified enrichment in genes associated with sugar metabolism within sweet corn kernels. Furthermore, the gene co-expression network constructed through WGCNA facilitated the identification of key modules associated with the quality of sweet corn. These modules encompass a range of genes closely related to biological processes such as sugar metabolism, growth and development. By integrating expression quantitative trait loci (eQTL) analysis with an investigation of the genetic regulation of malate and ubiquitin, our findings are further reinforced that the observed transcriptional disparities among sweet corn varieties primarily stem from differences in regulatory networks, as opposed to the modulation of isolated genes.

### 4.3 Prospects for sweet corn quality improvement

One of the significant challenges in sweet corn breeding is the presence of genetic limitations that can restrict the improvement of key traits such as sugar content, kernel texture, and disease resistance (Tracy et al., 2019; Hu et al., 2021a). To address these limitations, several innovative strategies have been proposed. A promising approach is to identify and aggregate favorable alleles for target traits within the existing sweet corn populations (Revilla et al., 2000; Wasuwatthanakool et al., 2022). However, due to significant differences from field corn at both the transcriptional and metabolic levels, improvements cannot be made by simply crossing to field corn. These problems have led to difficulties in improving the quality of existing varieties. Although some efforts have been made to improve sweet corn through molecular marker-assisted selection (MAS) using key genes related to quality and nutrition identified in field corn, these attempts have not resulted in significant improvements (Jompuk et al., 2020; Baveja et al., 2022). Another possible strategy is the modification of key genes using genome editing techniques such as CRISPR-Cas9 (Wang et al., 2022; Ahmar et al., 2023). Genome editing enables precise and targeted modifications to the sweet corn genome, providing a powerful tool for enhancing specific traits. However, it is important to note that genome editing is a complex process that requires a deep understanding of gene function and regulation (Zhang et al., 2021; Wang et al., 2022). MAS can be employed to integrate these genetic modifications into breeding programs. By combining these approaches, it is possible to overcome genetic limitations and achieve improvements in sweet corn quality.

The aggregation of favorable alleles and the targeted modification of key genes using genome editing techniques, coupled with the efficient integration of these changes through marker-assisted selection, hold great promise for the future of sweet corn breeding. These strategies could lead to the development of new varieties with enhanced flavor, texture, nutritional value, and disease resistance, meeting the growing demand for high-quality sweet corn worldwide.

## 5 Conclusion

Through analysis of gene expression, metabolite composition, and co-expression networks among different types of sweet corn, we uncovered key differences in sugar metabolism among various sweet corn varieties. These findings not only shed light on the molecular mechanisms underlying variations in sweet corn quality but also offer valuable insights for future improvement of sweet corn. These findings have significant practical implications for the breeding of sweet corn varieties with enhanced yield and quality.

## Data Availability

The original contributions presented in the study are publicly available. This data can be found at the National Center for Biotechnology Information (NCBI) using accession numbers PRJNA1146012 and PRJNA1145475.
